# Books and Authors

**Published:** 1910-11

**Authors:** 


### BOOKS AND AUTHORS

---

**Nephrocoloptosis.** A description of the nephrocolic ligament and its action in the causation of Nephroptosis. With the technic of the operation in Nephrocolopexy. By **H. W. Longyear**, M.D., Professor of Gynecology and Abdominal Surgery in the Detroit Post-Graduate Medical School; Clinical Professor of Gynecology in the Detroit College of Medicine; Gynecologist to Harper Hospital; Ex-president of the American Association of Obstetricians and Gynecologists. Octavo, pp. 251. With 88 illustrations and a colored frontispiece. Saint Louis: C. V. Mosby Co. 1910. (Cloth $3.00.

The author of this monograph believes that he has discovered in the nephrocolic ligament the principal etiologic factor in nephroptosis. Longyears's contention regarding the action of the nephrocolic ligament supports Glenard's dogma—"Enteroptosis without nephroptosis, but never nephroptosis without enteroptosis." Nephrocoloptosis admittedly is a widespread condition, not limited by race, nation, social status, or sex, hence its study is important, that the expression of its symptomatology may be interpreted with greater accuracy than has been done heretofore. We believe Longyear has made an important contribution toward solving this difficult problem.

The author first deals with the anatomy involved in the study of displaced kidney. In the course of his studies he discovered the nephrocolic ligament, of which he gives an elaborate and admirable description. He also gives directions for the dissection necessary to demonstrate the presence of the nephrocolic ligament, and shows by diagrams the relations of the kidney to the adjacent structures.

In the succeeding chapter is presented the etiology, dealing with the office of the nephrocolic ligament in pulling the kidney out of place, and of the combinations of conditions necessary to the displacement of the kidney ; also explaining why the right kidney is oftener displaced. The third chapter considers symptomatology, the intestinal manifestations being predominant. The author points out that jaundice and biliary disturbances are caused by traction on the duodenum. Diagnosis forms the subject of the fourth chapter, in which great stress is laid upon posture in making the examination. The dorsal and lateral decubitus are fully described and excellently illustrated. The *x*-ray is invoked as an important aid in the diagnosis.

The fifth chapter is assigned to the consideration of treatment. This, according to Longyear, must be mechanical in the main. Medicinal treatment must be directed principally to the colonic function. Topically, heat is the most efficient agent. The author's abdominal supporter is an ingenious contrivance, well adapted to many cases. But, in the majority of instances, operative measures are the most effective in bringing about permanent relief. The operation devised by the author fixes both kidney and bowel by utilising the nephrocolic ligament. Longyear describes and illustrates his technic in detail and makes valuable post-operative suggestions.

The sixth and last chapter presents reports of fifty-four cases that he has operated without mortality. The illustrations, 88 in number, are of a high order, depicting clearly the ideas of the author on each feature portrayed.

Dr. Longyear presented this subject, in part, as a president's address at the eighteenth annual meeting of the American Association of Obstetricians and Gynecologists held at New York, September 19, 20, and 21, 1905. He further developed the topic at subsequent meetings of the association, notably at Fort Wayne, in 1909, where he gave a stereopticon demonstration of its more perfected status. His work constitutes a distinct, lasting, and original contribution to the literature of nephrocoloptosis, and deserves, as it no doubt will receive, the careful attention of all abdominal surgeons.

---

**Progressive Medicine,** Vol. 12, September, 1910. A quarterly Digest of Advances, Discoveries and Improvements in the Medical and Surgical Sciences. Edited by **Hobart Amory Hare**, M.D., Professor of Therapeutics and Materia Medica in the Jefferson Medical College of Philadelphia, and **Leighton F. Appleman**, instructor in therapeutics at the same college. Philadelphia and New York: Lea & Febiger. (Per annum, paper bound, $6.00; cloth, $9.00.)

The contributors and titles in this number are William Ewart, London, diseases of the thorax and its viscera, including the heart, lungs, and blood vessels; William S. Gottheil, New York, dermatology and syphilis; Edward P. Davis, Philadelphia, obstetrics; and William G. Spiller, Philadelphia, diseases of the nervous system. Tuberculosis receives first attention by Ewart, but we were interested in his abstract on fetid bronchitis which, though brief, revived an old subject not much referred to in recent literature.

In Gottheil's abstract grain itch receives deserved attention and is well illustrated. Pellagra is touched upon, and new cases are reported, but its treatment has not been effective, its mortality still remaining high. Pregnancy eruptions are noticed and illustrated, while syphilis receives marked attention. New methods of diagnosis are weighed and assigned their appropriate places. The spirochete is admitted to be present in all syphilitic lesions and, though not yet obtainable in pure cultures, it is entitled to be regarded as an etiological factor in the disease. The Wassermann reaction is difficult to make, a well-equipped laboratory is needed for its demonstration, and an expert only can make it. Syphilis maligna is dealt with at length, and the illustrations of this form of the disease are excellent.

Davis devotes considerable space to obstetrics and has made interesting abstracts. Pregnancy and its complications is full of important comment, particularly the toxemia of pregnancy. The treatment of pernicious nausea by adrenalin, both internally and applied to the nasal mucous membrane, has given results in some instances. The absolute cause of eclampsia is still in doubt. Holland, London, has made extensive analysis, but the special eclamptic toxin has not been found, though the disease, in the majority of instances, is of toxemic origin. Obstetric surgery claims considerable attention, though we miss reference to Porter's able paper on cesarean section read before the American Association of Obstetricians and Gynecologists at Fort Wayne, September, 1909. (Am. Jour. Obstetrics, November, 1909. Trans. Am. Association Obstetricians and Gynecologists, Vol. XXII. 1909.)

The newborn forms the subject of considerable valuable comment, among which is an analysis by Dorman on the causes of death of the viable fetus before labor.

Spiller makes a digest of diseases of the nervous system, including brain tumors, poliomyelitis, fractures of the vertebræ, epilepsy, and many other topics of greater or less importance. This number is full of interesting material and will prove of special value to the general practitioner.

**A Manual of Operative Surgery.** By **Sir Frederick Treves**, Bart., G.C.V.O., C.B., LL.D., F R C S, Sergeant-Surgeon to H.M the King, and **Jonathan Hutchinson**, F.R.C.S., Surgeon to the London Hospital. Third edition. In two octavo volumes. Volume II, 820 pages, with 302 engravings, and 8 full-page plates. Lea & Febiger, Publishers, Philadelphia and New York, 1910. (Half-morocco, $6.50, net.)

Mr. Hutchinson, the associate of Sir Frederick Treves in the authorship of the manual, makes apology for the delay in the publication of this volume, which he says was due, at least in large part, to the amount of time and labor incident to the revision of the whole work because of the numerous additions and alterations made to it. This volume contains parts three and four, and a number of excellent plates, besides many new illustrations in the text. The third part deals with operations on the neck and spine, while the fourth part presents operations on the thorax and breast. The first two chapters in the third part relate to operations on the skull and brain and to operations on the middle ear and mastoid antrum, and have been revised, even largely rewritten by Mr. A. J. Walton, while the chapter on tendon suture and tendon grafting is entirely his work.

It is a genuine pleasure to possess this completed treatise, for it places in our hands,—in the hands of the medical profession of America,—the experience of two of the greatest of British surgeons. It is pleasant to be able to refer to plastic operations, as set forth by text and illustration in this work. Treves and Hutchinson cling to the traditions of surgery and present the classic operations of the whole surgical domain while, at the same time, adopting every modern method. Every surgeon will feel great satisfaction in keeping this work close at hand for convenient reference.

